# Book Reviews

**Published:** 1875-12

**Authors:** 


### Book Reviews.

[NOTE. — All works reviewed in the pages of the CHICAGO MEDICAL JOURNAL AND EXAMINER may be found in the extensive stock of W. B. KEEN, COOKE & CO., whose catalogue of Medical Books will be sent to any address upon request.]

THE HEART AND ITS DISEASES: WITH THEIR TREATMENT. By *J. Milner Fothergill, M.D., M.R.C.P.* London: H. K. Lewis.

This is a volume of 382 pages, neatly printed on tinted paper, and free from those offensive advertisements which occupy such a considerable space in many books.

Following Bernard, Cyon, Rutherford, Pettigrew, Brunton, Richardson, Quain, Geo. Johnson, Traub, and others, the author has attempted to place the "Heart and its Diseases" before the profession under the full light of recent investigations.

Though we cannot accept some of the theories advanced, we must respect the manner in which they are given, and the names of the investigators who support them. Some things which seem probable to the author seem very improbable to us ; for instance, he thinks that hysteria, one of the causes of palpitation, is always the result of ovarian congestion or chronic ovaritis. We think this an error, though, of course, we agree that palpitation may be produced by irritation of the sexual organs.

The description of the structure of the heart and its nerve supply is doubtless more nearly correct than that found in older works. With regard to the disputed question, as to the causes of the first sound of the heart, the author believes that there are two principal factors viz., contraction of muscular fibre of the ventricular walls, and closure of the auriculo-ventricular valves. Two lesser factors are given, viz., the apex beat against the chest wall, and the rushing of blood through the aortic and pulmonic orifices.

In some recent publications, muscular contraction *per se* is said to have no part in the production of the heart sounds, and the majority of the profession seem of this opinion ; yet we think any auscultator may satisfy himself of the correctness of the author by a simple experiment. As we listen over the ball of the thumb, where the skin will not move beneath the stethoscope, we hear with each contraction of the subjacent muscles a sound similar to the first sound of the heart. A like sound, though more intense, may be obtained over the fore-arm while the fingers are being flexed.

It is important to appreciate this fact, for the muscular element of the first sound varies in intensity directly with the varying strength of the ventricular walls.

The author's remarks upon the character of the pulse, as a means of diagnosis, are worthy of attention ; and his descriptions of some of the signs and symptoms of cardiac diseases are uncommonly clear.

In place of the common belief that little can be done for patients suffering with disease of the heart, the author holds that many cases may be greatly benefited, and some cured, by appropriate remedies. Briefly, his treatment is as follows: First, whenever possible, remove the cause. If this is found in the patient's business or habits, these must be changed; if in the rheumatic or gouty diathesis, this must be combated; if in the kidneys, as in Bright's disease, the blood must be purified through the other emunctories.

Hypertrophy is always conservative, and, therefore, of itself requires no treatment. When the heart begins to fail, remedies are needed which strengthen its pulsations, and thus tend to induce hypertrophy. For this purpose digitalis is specially recommended, and after it belladonna, caffeine and squill; at the same time iron, quinine and good diet may be required. In the latter stages of heart failure, much may be done by way of palliation, though we cannot hope for permanent relief.

The subject of prognosis is treated of, in this, more satisfactorily than in most kindred works.

Several rare or obscure diseases of the heart are described; such as ulcerative endocarditis, fatty infiltration, connective tissue hypertrophy, irritable heart, syphilitic gummata, etc.

The atheromatous process, its causes and effects, are well described, and a short but interesting chapter has been introduced on malformations of the heart. The thirteenth is a chapter of special interest, treating of combined heart and kidney disease.

Having called our attention to the fact that imperfect action of the kidneys allows an accumulation of urinary salts in the blood, the author goes on to show that these salts, acting as a poison, affect, first, the vaso-motor nerves, and through them cause spasm of the arterioles. Repeated spasm of these vessels causes thickening of their walls, and a corresponding diminution of their calibre, which, by impeding the flow of blood, necessitates an increased action of the heart, and this sooner or later induces hypertrophy.

Cardiac hypertrophy, though a conservative process, is apt to be followed by a train of unfortunate consequences. At first no ill effects are noticed. The augmented force of each pulsation produces extreme distension of the elastic arteries, and this is followed by increased recoil, which, by forcing a larger amount of blood through the coronary arteries, supplies the ventricular walls with the increased nourishment demanded. Eventually, however, strain upon the arteries induces atheroma of their walls; they become rigid, and thus offer a new impediment to the flow of blood, while at the same time they are so weakened that the laboring heart is liable to cause rupture of their fragile coats. Rupture is most likely to occur in the thin walled arteries within the encephalon, therefore, in this condition, we have great liability to apoplexy. Diminished elasticity of the arteries lessens their recoil, in consequence of which the coronary blood-vessels are imperfectly filled, and the heart walls suffer for want of nutriment. As results—hypertrophy, the necessity for which is constantly increasing, ceases; atrophy follows, and soon dilatation; and with dilatation come all the baneful effects of venous congestion, and imperfect oxygenation of the blood.

In this chapter also are shown the effects of renal disease on other organs, as the brain, air passages, alimentary canal and skin.

The author has shown, the manner in which disease of the heart may cause Bright's disease; and how these diseases act and react upon each other, under the constant effort of nature to maintain an equilibrium between the various organs of the body.

On a fly-leaf half a dozen mistakes are pointed out, but we cannot understand why these are mentioned while the multitude remain unnoticed. It has never been our fortune to read a book so filled with grammatical faults and typographical errors. Involved and obscure sentences are constantly occurring, to the great annoyance of the reader. In some of these the author is made to say the reverse of what he evidently intended, and in others the thought is so hidden by the extraordinary punctuation and construction that it would be difficult for any one, unfamiliar with the subject, to find it. Plural are used for singular verbs, and *vice versa*. The tenses are carelessly employed, and pronouns are separated so far from their antecedents that the relation can hardly be traced. We quote as illustrations: "The muscles of the body largely cross the arteries, and, when in action, constricts them and impede the flow of blood through them." "It is often best given after food, and is then digested with the food, in the shape of drops, pill, or powder." The obscurity of many paragraphs is well illustrated by the last sentence in the book, which, from its length and construction, much resembles the style of "Mrs. Flinching," in Dickens' *Little Dorrit*.

Many of these errors must be chargeable to the printer and proof reader. Doubtless others result from the author's desire to avoid verbosity; if so, we can almost excuse him, for the work is condensed, and yet complete.

As a whole, this is a valuable book, with substantial merits and non-essential faults; yet we are much surprised at its errors, for we had supposed that such shortcomings were confined to American authors and publishers. Especially in England, where they do not deign to recognize diplomas from our medical schools, we expected to find little less than perfection in medical publications. When such an imperfect book is written by a gentleman with M.D., M.R.C.P., attached to his name, we are led to ask, in what does the superiority of a foreign education consist?

E. F. I.

VISION: ITS OPTICAL DEFECTS AND THE ADAPTATION OF SPECTACLES. With 74 Illustrations on Wood. By *C. S. Fenner, M.D.* Philadelphia: Lindsay & Blakiston.

The literature of Medicine possesses in "Helmholtz's Physiological Optics" and "Donder's Anomalies of Refraction and Accommodation," two works which treat of vision and its optical defects in a way that can hardly be surpassed. Both books, however, containing so much that is unpalatable for a reader not familiar with the higher mathematics, have not found so large a circulation in the profession as they deserve for their ingenious composition and their interesting contents. For this reason the author has endeavored "to give, in a concise and popular, yet comprehensive form, a resumé of our present knowledge of physiological optics and the defects of the eye as an optical instrument."

The book is divided into three parts and an appendix. The *first part*—Physical Optics (pages 17 to 68) is a short abstract of the properties of light and its physical laws; it is evidently intended to give the reader such preliminary information as he needs in order to understand the other parts of the book. The author would have done better had he omitted such subjects as Fluorescence, Phosphorescence, Spectral Analysis—interesting though they may be—and by enlarging upon those laws which have a direct bearing on the optical properties of the eye.

The *second part* (pages 68 to 168) is devoted to the discussion of "Dioptrics," "Visual Sensations" and "Visual Perceptions." The composition of this portion of the book has materially suffered through the inability of the author himself to write and revise the manuscript on account of a serious affection which befell his eyes. Too brief in some important points (dioptrics, ophthalmoscope), too lengthy in some immaterial questions (shape of spectacles), it is not arranged in that systematic order most intelligible to the learner. Confusion creates confusion.

The *third part* (pages 168 to 284) treats in a plain and comprehensive way of the "Errors of Refraction," (Hypermetropia, Myopia, Astigmatism) and the "Defects of Accommodation," (Presbyopia, Aphakia). But we are greatly surprised at the author's absolute silence in regard to paralysis and spasms of the accommodation, the action of atropia and calabar. He dismisses hurriedly the determination of hypermetropia and its degree, by means of the ophthalmoscope. Does he not deem it necessary to control the result obtained by the test-glasses, by an ophthalmoscopic examination, in order to guard himself against mistakes? Has he not met with persons who by the test-glasses are apparently myopic but by the ophthalmoscope are actually hyperopic? He does not allude to these phenomena at all.

The "Appendix" gives some plain instructions for the adaptation of spectacles to persons so situated that they cannot well obtain competent professional advice. We do not think much of this popular advice; it will do neither harm nor good. And in spite of it the traveling optician will always find a good market for his "pebbles"!

The wood cuts are well executed, and print and paper are good. In fine, this book, like the majority of these "concise and popular" treatises, cannot satisfy the reader. Incomplete as they are, they can but stimulate the desire of the student for more knowledge of this rich and interesting field. But if it awaken this interest it accomplishes a great deal and fulfills its task.

Lectures on Diseases of the Nervous System. By *Jerome K. Baudery, M.D., etc.* Philadelphia: J. B. Lippincott & Co.

This book claims nothing original "as to its facts or as to its theories." It professes to be a course of lectures recently delivered by Prof. Baudery before the students in the Missouri Medical College, or rather, it is made up from the notes of such lectures taken down "by V. Biart, M.D., his former pupil." Strictly speaking, it should have been entitled "Prof. Baudery's Scrap-book," in which "the views of Maudsley, Van der Kolk, Blandford and Gray, of Utica, will be frequently recognized" along with "the classic representations and literary photographs of disease by the immortal Trousseau, the forcible diagnostic elucidations of Da Costa, and the beautiful and interesting theories of the late Dr. Bentley Todd." The preface contains the names of not less than thirty-five different authors to whose works he is indebted for the major part of his own. This recognition of authority is honorable, and would be highly praiseworthy had there been more cause for it. But the list, unfortunately, contains not one of the names of the most celebrated French and German teachers who have recently written upon the subject of nervous diseases. To attempt the compilation of a treatise concerning any scientific subject without an acquaintance with the most recent works of French and German specialists in the original, is an unpardonable sin. The bookmaker who forages only among English books and English translations is likely to find himself ten years behind the times. This, we fear, is true of too much that is palmed off upon the unsuspicious medical student. It certainly is true in the present instance. Since, then, we are dealing with a compilation of this sort, rather than with an original work, it would be hardly fair to single out for objection any particular passages which may admit of question. As the lawyer said, when the judge overruled all his special objections, "we object to the whole proceeding."

Such books are not needed by practitioners of medicine—their necessities demand a wider range of reference. They are worse than useless to medical students, because they tend to beget a cursory, slip-shod method of study, and, moreover, do actually serve as a barrier between the mass of students and the really good books which they ought to find in their hands. When the average graduate has purchased a volume like this, perhaps the production of a favorite professor, it will be a long time before he buys another — even though ten-fold more valuable and recent than the one in which he has invested his funds.

This book will secure for its author a certain kind of reputation, but its effect upon that portion of the profession who may accept it as their *vade mecum* will be nothing less than cerebral anæmia, and mental paresis. H. M. L.

On Poisons in Relation to Medical Jurisprudence and Medicine. By *Alfred Swayne Taylor, M. D., F. R. S.* Third American, from the Third and Thoroughly Revised English Edition. Philadelphia : Henry C. Lea.

"Taylor on Poisons" is so well known to English speaking physicians that an extended notice of a new and thoroughly revised edition is not needed. The work has been for some time out of print, because the author has proferred to bestow upon it the care and attention needed to make it fully equal to the present aspects of toxicological science, rather than to issue a mere reprint of a former edition. The chapters omitted have been fully replaced by new matter in accordance with the needs of to-day, keeping the work strictly practical. As a manual for the student and practitioner we do not hesitate to pronounce this the best of its kind in our language.

It is pleasant to find the investigations of American physicians quoted so fully by a standard authority of conservative old England. H. P. M.

Die Resultate der Gelenkresectionen im Kriege. Nach eignen Beobachtungen. Von *E. Bergmann*, Professor d. Chirurgie in Dorpat. Giessen, 1874. 4° mit 20 Tafeler albertotypie und einer lithographirten Tafel.

The Results of the Excisions of Joints during the War. By *E. Bergmann*, Professor of Surgery in Dorpat. Giessen, 1874.

This is a very interesting account of the excisions of joints performed by Professor Bergmann during the Franco-German war, in the hospitals at Mannheim and Karlsruhe. He reports nine excisions of the elbow (two fatal results), fifteen excisions of the shoulder (three fatal results), and eleven of the ankle joint (two fatal results).

The history of each case is given very extensively, and what adds very much to the value of the book, the ultimate condition of the limb when examined two and three years after the operation, is carefully recorded and demonstrated *ad oculos* in twenty photographic plates. A lithographic plate contains the drawings of a number of the excised bones.

---

